# Screening differentially expressed proteins to distinguish thymoma (B1 and B3) from thymic cysts based on tandem mass tag (TMT) technology

**DOI:** 10.1186/s13019-024-03114-x

**Published:** 2024-10-21

**Authors:** Jingwei Shi, Rusong Yang, Xin Chen, Yan Wang, Ye Shi, Yongsheng Wang, Zhengcheng Liu

**Affiliations:** 1 Department of Thoracic Surgery, Affiliated Hospital of Medical School, Nanjing Drum Tower Hospital, Nanjing University, Nanjing, China; 2 Department of Cardiology, Affiliated Hospital of Medical School, Nanjing Drum Tower Hospital, Nanjing University, Nanjing, China; 3 Department of Anesthesia, Affiliated Hospital of Medical School, Nanjing Drum Tower Hospital, Nanjing University, Nanjing, China; 4 Department of Thoracic Surgery, Affiliated Nanjing Brain Hospital, Nanjing Chest Hospital, Nanjing Medical University, Nanjing, China; 5 Department of Respiratory Medicine, Affiliated Hospital of Medical School, Nanjing Drum Tower Hospital, Nanjing University, Nanjing, China

**Keywords:** Thymic cyst, Thymoma, Proteomics, LGALS3BP

## Abstract

The therapeutic approach to thymic cysts remains a subject of controversy. Predicted biomarkers for identifying thymic cysts and thymoma (THYM) are crucial. In this research, patients diagnosed with thymic cysts (MTC, *n* = 6) and thymoma (B1, *n* = 6; B3, *n* = 6) were enrolled. Proteins of superior quality were subjected to TMT labeling and UPLC-MS, and differentially expressed proteins (DEPs) were identified. Gene Ontology (GO), Kyoto Encyclopedia of Genes and Genomes (KEGG), and protein-protein interactive network analyses were applied to the DEPs. Some key differentially expressed genes(DEGs) were corroborated through GEPIA 32. The pan-cancer expression levels of key DEGs remarkably linked with prognosis were determined utilizing The University of ALabama at Birmingham CANcer data analysis Portal (UALCAN). Eventually, 49 DEPs were identified in the B1 vs. MTC comparison (17 upregulated and 32 downregulated), 27 in the B3 vs. MTC comparison (8 upregulated and 19 downregulated), and 38 in the B3 vs. B1 comparison (9 upregulated and 29 downregulated). IL13RA1 (down), galectin-3 binding protein (LGALS3BP)(up), PRCSH (down), C3 (down), MXRA5 (down), TNN (down), CFHR1 (down), SUN3 (down) were jointly altered in both B1 vs. NZ and B3 vs. NZ. GEPIA validated that LGALS3BP was significantly upregulated in thymoma patients. In conclusion, LGALS3BP might be an essential biomarker to identify thymoma from the thymic cyst.

## Introduction

Thymic cysts represent one among several types of mediastinal cysts found in the chest, with the etiology of mediastinal cysts being diverse and complex. Most of them are congenital, and some are acquired, but the root causes of thymic cysts are not known. It is believed that thymic cysts are innate as they are found on the development line of the thymus, such as in the neck and mediastinum. Thymic cysts account for approximately 1% of mediastinal tumors and cysts [1, 2]. Preoperative diagnosis of thymic cysts poses challenges. Due to their location and morphological characteristics, thymic cysts can be misdiagnosed as thymomas, while their calcification may contribute to their being misdiagnosed as teratomas. A thymic cyst, situated in proximity to the pericardium, may be misdiagnosed as an aortic aneurysm, particularly in cases where conductive pulsations are observed on fluoroscopy. The definitive identification of such cases requires surgical exploration [3].

Thymoma is one of the most prevalent forms of tumor of the anterior mediastinum. In terms of the WHO classification method, thymoma was classified into five major subtypes (A, AB, B1, B2, and B3), including thymomas containing spindled neoplastic epithelial cells (A, AB) and thymomas having epithelioid neoplastic epithelial cells (B1-3) [4]. Type B thymoma is subdivided into B1, B2, and B3 as per the proportion of benign and malignant lymphocytes. Presently, the treatment of mediastinal occupation remains controversial. Several experts advocate for surgical resection as a proactive approach to addressing the challenges associated with diagnosing thymic cysts preoperatively. This not only ensures the removal of the lesion but also facilitates an accurate diagnosis [5]. Thymic cysts, characterized by distinct borders and attachment to the thymus, exhibit ease of peeling during surgical procedures. However, some proponents believe that a thymic cyst can be cured by percutaneous fine needle puncture, provided a precise determination of its location and CT imaging characteristics is achieved [6]. For patients lacking a definitive diagnosis of thymic cysts, surgical intervention becomes imperative to achieve the dual objectives of accurate diagnosis and effective treatment. This is especially crucial when the possibility of thymoma with cystic change and hydatid cysts cannot be entirely ruled out. The surgical techniques for excising thymic cysts include median sternal incision, anterolateral incision, and posterolateral incision. Video-assisted thoracoscope can also be used to perform the operation. Diagnostic imaging techniques are essential for evaluating patients with thymoma; specifically, tomography studies are necessary to evaluate tumor spread into the mediastinum; within these imaging modalities, computed tomography (CT), magnetic resonance imaging (MRI), and positron emission tomography (PET) embody the cutting-edge diagnostic technologies for such tumor lesions. However, CT imaging has limitations in accurately evaluating these lesions. Misinterpretation of chest CT as indicating thymoma leads to unnecessary thymectomy procedures in 43.8% of cases [7]. Although MRI is not frequently employed in the assessment of thymic tumors, it offers unique benefits in certain circumstances. For instance, it aids in differentiating solid from cystic lesions when CT results are ambiguous, assesses the cystic or necrotic components of a mass, evaluates the septum enhancement within cystic lesions, and detects subtle areas of local infiltration. Although biopsies may offer assistance in such cases, a non-invasive preoperative diagnosis is urgently required.

Proteomics encompasses the study of proteins on a large-scale level, focusing on their characteristics, such as protein-protein interaction and protein expression level, to understand disease occurrence, cell metabolism, and other processes on the protein level. The study of proteomics can aid in the identification of predicted biomarkers for diseases and provide a theoretical foundation and solutions for elucidating and combating numerous disease mechanisms [8]. This technology has been used to identify biomarkers in nearly all cancers [9, 10].

In this study, biomarkers of B1 and B3 thymoma and thymic cysts were identified through proteomics. The objective of this research is to identify diagnostic biomarkers that can be utilized in clinical practice to distinguish mediastinal occupation.

## Materials and methods

### Recruitment of participants

The approval for this research was granted by the institutional review board of the Nanjing Chest Hospital (number of ethics approval: 2017-KL002-03). Additionally, all participants provided signed informed consent. Patients with thymic cysts (MTC, *n* = 6) and thymoma (B1, *n* = 6; B3, *n* = 6) hospitalized in Nanjing Chest Hospital (Nanjing, China) were included in this study. The criteria for inclusion were (1) age 18–70; (2) American Society of Anesthesiologists (ASA) grade of I-II; (3) Masaoka stage 1–2; (4) no preoperative treatment. The exclusion criteria were (1) patients not suitable for surgery; (2) Thymoma with myasthenia gravis; (3) pathologic diagnosis other than thymic cyst or thymoma (type B1 and B3).

### Protein isolation and SDS-PAGE

Tumor tissues from patients were isolated, segmented into small pieces, and immersed in liquid nitrogen. Prepared tissues were homogenized and digested by SDT Lysis Buffer (cat: #XY91029). Protein concentrations were measured by BCA kit (P0012; Beyotime). Subsequently, 20 µg of each protein sample mixed with 6X loading buffer solution underwent boiling for 5 min. Separation was performed by utilizing 12% sulfate sodium salt-polyacrylamide gel electrophoresis (SDS-PAGE) at 250 V for 40 min. Proteins were stained by Coomassie brilliant blue. Samples with a total amount ≥ 500 µg and completed protein lines were used for further study.

### TMT labeling

Protein samples underwent enzymatic digestion by the Filter-aided proteome preparation (FASP) method [11], followed by peptide quantification (OD280). Subsequently, the peptides underwent labeling utilizing the corresponding TMT labeling reagents and were subjected to analysis through high-precision mass spectrometry.

### Ultra-performance liquid chromatography–MS (UPLC–MS)

An easy nLC system was used to examine the collected fractions. UPLC-MS was performed according to the methodologies outlined in a previous study [12]. Briefly, buffers A and B were 0.1% formic acid aqueous and 0.1% formic acid acetonitrile (acetonitrile concentration 80%). Samples were separated at a flow rate of 300 ml/min. After separating the chromatography column, samples were assessed by employing the Q Exactive mass spectrometer (Thermo Fisher Scientific). Wang et al.. outlined the key parameter settings for the Q Exactive mass spectrometer.

### Protein identification

A high-resolution mass spectrometer Q Executive plus (Thermo Fisher Scientific) was utilized for quantitative proteomic analysis of TMT. Proteome Discoverer 2.1 (Thermo Fisher Scientific) was utilized to convert the initial map file (raw file) produced by Q Executive Plus into Mgf. file. Subsequently, the Mgf. file was submitted to the MASCOT2.6 server for the database retrieval (Uniprot_HomoSapiens_20386_20180905). Afterward, Mascot 2.6 and Proteome Discoverer 2.1 were utilized for database checking and quantitative protein identification according to FDR < 0.01. Proteins with *P* < 0.05 and fold changes *> 1.1* (upregulated) or *< 0.90* (downregulated) were considered as differentially expressed proteins (DEPs).

### Bioinformatics analysis

For DEPs, Gene Ontology (GO) analysis (http://geneontology.org/) and Kyoto Encyclopedia of Genes and Genomes (KEGG) analysis (https://www.kegg.jp/) were conducted, with adjusted *P*-value *< 0.05*.

### Protein-protein interaction (PPI) analysis

The PPI analysis on DEPs in B1 vs. MTC, B3 vs. MTC, and B3 vs. B1 was carried out utilizing STRING28 (v10.0, http://www.string-db.org/), with a parameter threshold set at 0.4 (medium confidence).

### Key differentially expressed gene (DEG) expression validation

The key DEGs within the module underwent expression validation and survival analysis employing GEPIA 32. Moreover, the pan-cancer expression patterns of key DEGs, which were remarkably linked to prognosis, were investigated utilizing The University of ALabama at Birmingham CANcer data analysis Portal (UALCAN) (http://ualcan.path.uab.edu/index.html) and The Cancer Genome Atlas (TCGA).

### Statistical analysis

Statistical analysis was conducted with the aid of Graphprism 8.0, with data presented as mean ± SEM. The comparison of averages between the two groups was performed utilizing an independent t-test, while a two-tailed Fisher’s test was employed to assess GO and pathway. A *P*-value of *< 0.05* was deemed statistically significant.

## Results

### Baseline features of the participants

The average age was 55.8 years in the B1 group, 51.0 years in the B3 group, and 53.8 years in the MTC group. No variations were observed in age or gender (all *P* > 0.05). Details are provided in Table 1.

**Table 1:** Baseline characteristics of the participants. (ASA: American society of anesthesiologists; FEV1: forced expiratory volume in 1 s)

		B1 group(*n* = 3)	B3 group(*n* = 3)	MTC group(*n* = 3)
Gender	Male	4	3	3
	Female	2	3	3
Age (mean)		55.8	51.0	53.8
Smoking History (no. of smokers)		3	3	3
FEV1(L)		2.73	2.71	2.90
Body mass index (kg/m^2^) (mean)		24.0	23.6	24.2
ASA status class				
I		6	5	5
II		0	1	1

### TMT-based proteomics identified 114 DEPs

**Fig. 1:**
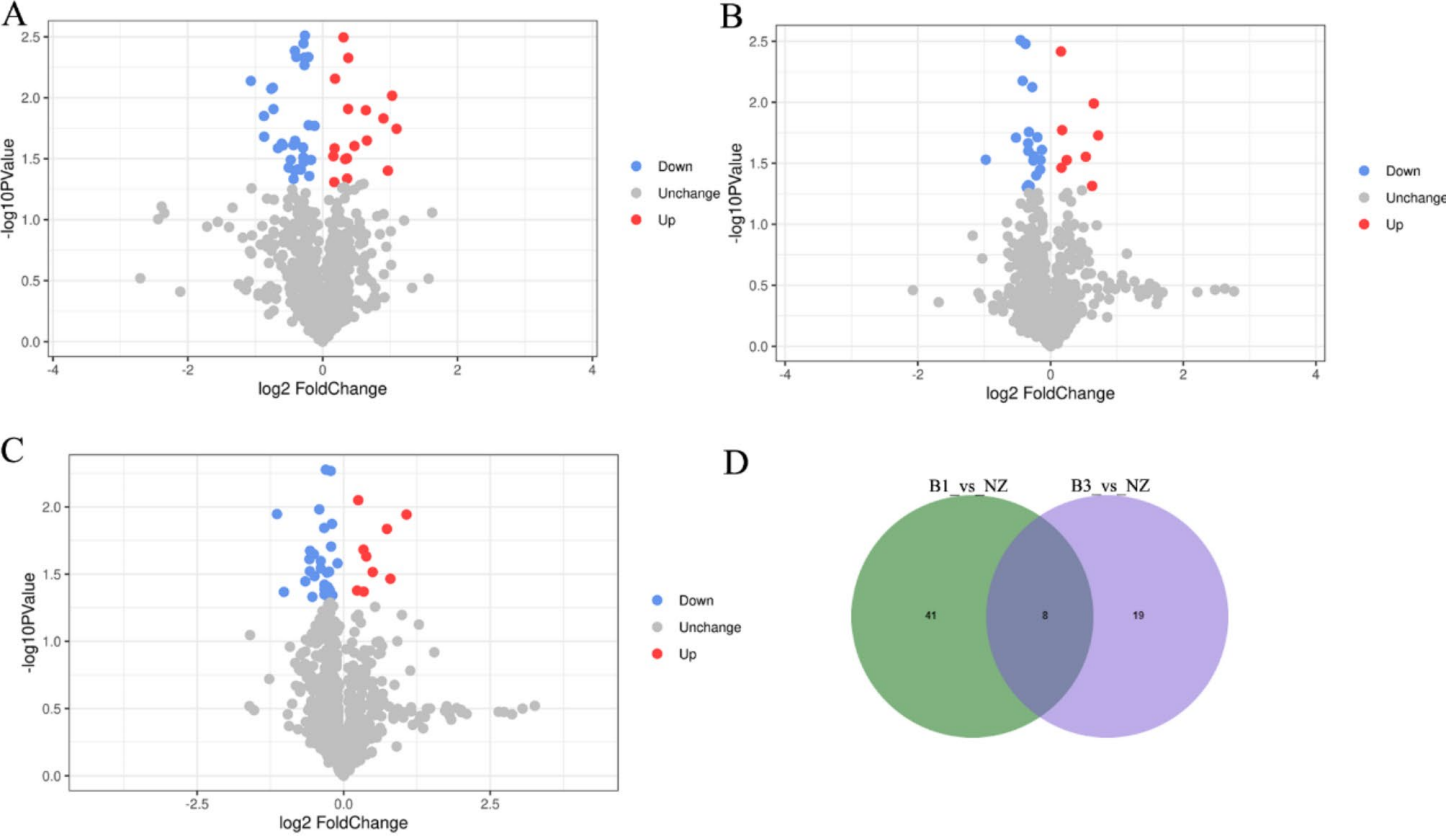
Volcano map of DEPs in B1 vs. MTC, B3 vs. MTC, and B3 vs. B1 comparisons. *Note* B1 vs. MTC, represents thymic cyst compared with B1 thymoma cancer. B3 vs. MTC, represents thymic cyst compared with B3 thymoma cancer. B3_vs_B1, represents B1 thymoma cancer compared with B3

In the serum of individuals with thymoma (B1 and B3) and thymic cysts, DEPs were identified by employing TMT-based proteomics to investigate the potential mechanisms underlying thymoma pathogenesis. A collective count of 49 DEPs was screened in the comparison between B1 and MTC (17 upregulated and 32 downregulated), 27 in B3 and MTC (8 upregulated and 19 downregulated), and 38 in B3 and B1 (9 upregulated and 29 downregulated). Figure 1 A-C depicts the volcano plot illustrating the DEPs across the three comparisons. GSTP1, IGHV1-18, F7, ANG, IL13RA1, AMBP, SERPINB3, SRI, SHISA5, and LGALS3BP emerged as the most significantly dysregulated DEPs in the comparison between B1 and MTC. Meanwhile, EPHX4, IL13RA1, PMFBP1, TIMD4, AHCYL1, IGKV4-1, LGALS3BP, PRKCSH, APOF, and F9 exhibited significant dysregulation in the B3 vs. MTC comparison. Additionally, MGAT1, IGF2R, INHBC, BST1, DEFA1, MBL2, AZGP1, AMY1A, APOF, and RNASET2 displayed significant dysregulation in the B3 vs. B1 comparison.(Table 2). After comparisons, 8 DEPs were determined to be differentially expressed in both B1 vs. MTC and B3 vs. MTC (Fig. 1D): IL13RA1 (down), LGALS3BP (up), PRCSH (down), C3 (down), MXRA5 (down), TNN (down), CFHR1 (down), SUN3 (down).

**Table 2:** The top dysregulated DEPs in B1 vs. MTC, B3 vs. MTC, and B3 vs. B1 comparisons

Accession	Gene name	Regulation	*p*-value
**B1 vs. MTC**			
P09211	GSTP1	DOWN	0.00309139
A0A0C4DH31	IGHV1-18	UP	0.003198779
P08709	F7	DOWN	0.003595216
P03950	ANG	DOWN	0.004119949
P78552	IL13RA1	DOWN	0.004630202
P02760	AMBP	DOWN	0.004635472
P29508	SERPINB3	DOWN	0.004681172
P30626	SRI	UP	0.004713567
Q8N114	SHISA5	DOWN	0.005404984
Q08380	LGALS3BP	UP	0.006979299
**B3 vs. MTC**			
Q8IUS5	EPHX4	DOWN	0.003095
P78552	IL13RA1	DOWN	0.003324
Q8TBY8	PMFBP1	UP	0.003829
Q96H15	TIMD4	DOWN	0.006676
O43865	AHCYL1	DOWN	0.007515
P06312	IGKV4-1	UP	0.01023
Q08380	LGALS3BP	UP	0.016919
P14314	PRKCSH	DOWN	0.017522
Q13790	APOF	UP	0.018709
P00740	F9	DOWN	0.019351
**B3 vs. B1**			
P26572	MGAT1	DOWN	0.005284316
P11717	IGF2R	DOWN	0.005403522
P55103	INHBC	UP	0.008921613
Q10588	BST1	DOWN	0.0104325
P59665	DEFA1	DOWN	0.01130518
P11226	MBL2	UP	0.01140585
P25311	AZGP1	DOWN	0.01339033
P04745	AMY1A	DOWN	0.01435306
Q13790	APOF	UP	0.01458595
O00584	RNASET2	DOWN	0.01971991

*Note* B1 vs. MTC represents B1 thymoma compared to thymic cyst. B3 vs. MTC represents B3 thymoma compared to thymic cyst. B3 vs. B1 represents B3 thymoma compared to B1 thymoma

### Bioinformatics analysis

**Fig. 2:**
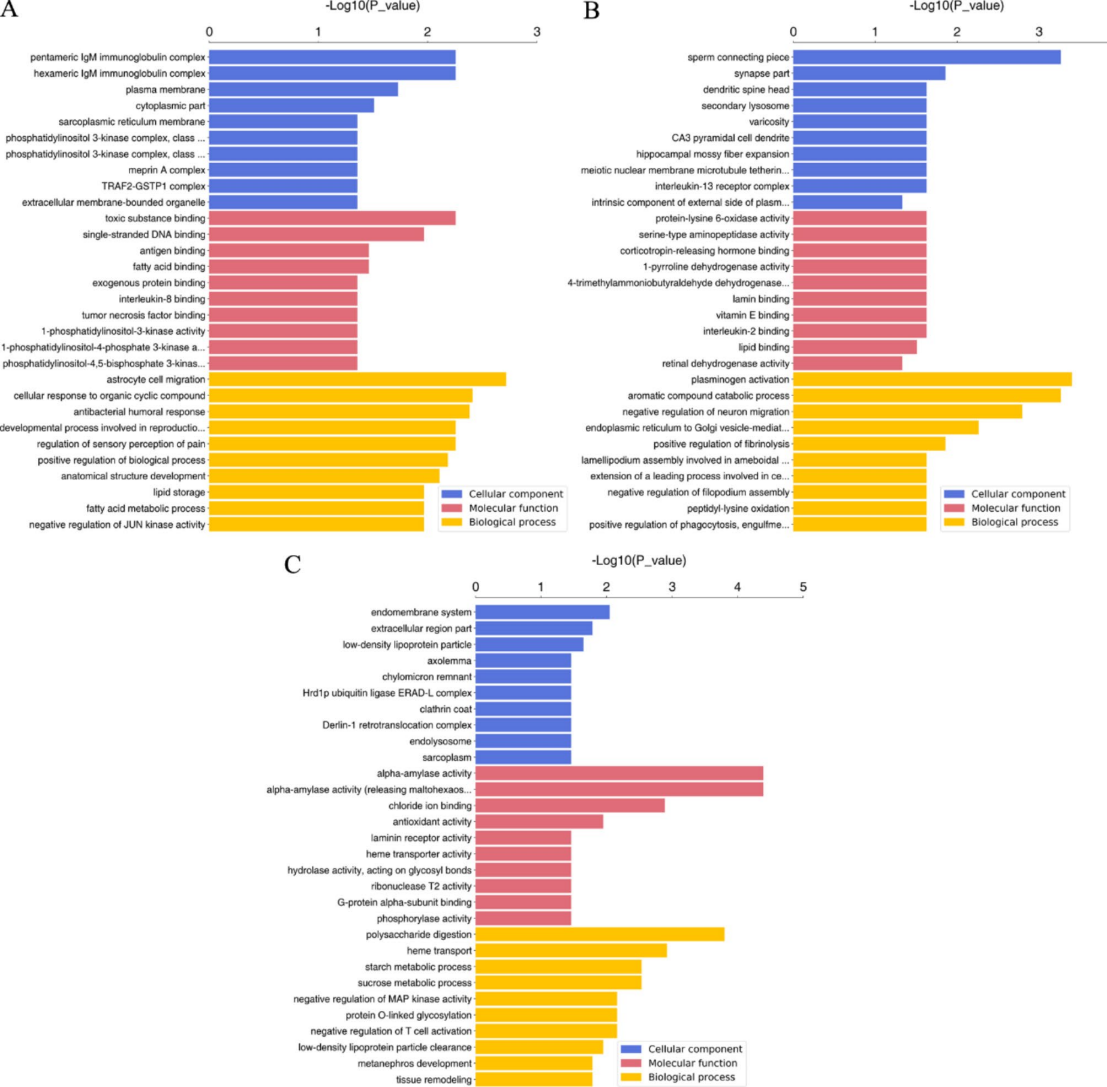
The GO analysis of DEPs in B1 vs. MTC (**A**), B3 vs. MTC (**B**), and B3 vs. B1 (**C**) comparisons

To find out the biological functions of the DEPs, GO enrichment analysis was conducted. The GO analysis revealed the involvement of these DEPs in diverse fundamental biological processes and the facilitation of numerous molecular functions. Comparison was made between the significantly altered GO terms. The DEPs in B1 vs MTC were predominantly enriched in ‘’astrocyte cell migration’’, ‘’cellular response to organic cyclic compound’’, ‘’antibacterial humoral response’’, ‘’toxic substance binding’’, ‘’pentameric IgM immunoglobulin complex’’, ‘’hexameric IgM immunoglobulin complex’’, ‘’developmental process involved in reproduction’’, ‘’positive regulation of biological process’’, ‘’regulation of sensory perception of pain’’, and ‘’anatomical structure development’’ (Fig. 2A). The DEPs in B3 vs MTC were predominantly enriched in ‘’plasminogen activation’’, ‘’sperm connecting piece’’, ‘’aromatic compound catabolic process’’, ‘’negative regulation of neuron migration’’, ‘’synapse part’’, ‘’endoplasmic reticulum to Golgi vesicle-mediated transport’’, ‘’positive regulation of fibrinolysis’’, ‘’dendritic spine head’’, ‘’lamellipodium assembly involved in ameboidal cell migration’’, and ‘’extension of a leading process involved in cell motility in cerebral cortex radial glia guided migration’’ (Fig. 2B). The DEPs in B3 vs B1 were mainly enriched in ‘’alpha-amylase activity’’, ‘’alpha-amylase activity (releasing maltohexaose)’’, ‘’polysaccharide digestion’’, ‘’heme transport’’, ‘’chloride ion binding’’, ‘’starch metabolic process’’, ‘’sucrose metabolic process’’, ‘’negative regulation of MAP kinase activity’’, ‘’protein O-linked glycosylation’’, and ‘’negative regulation of T cell activation’’ (Fig. 2C).

**Fig. 3:**
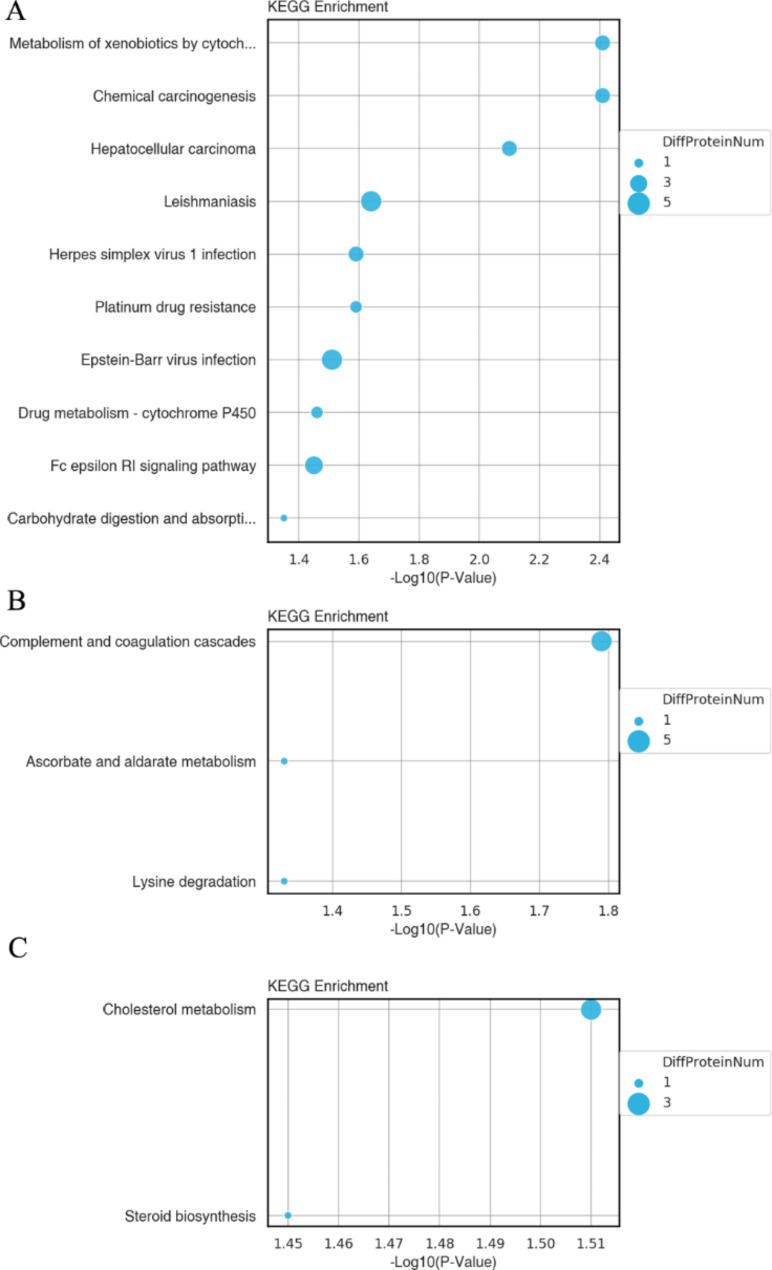
The KEGG analysis of DEPs in B1 vs. MTC (**A**), B3 vs. MTC (**B**), and B3 vs. B1 (**C**) comparisons

Furthermore, the KEGG analysis suggested that the DEPs in B1 vs MTC were predominantly enriched in ‘’Metabolism of xenobiotics by cytochrome P450’’, ‘’Chemical carcinogenesis’’, ‘’Hepatocellular carcinoma’’, ‘’Leishmaniasis’’, ‘’Herpes simplex virus 1 infection’’, ‘’Platinum drug resistance’’, ‘’Epstein-Barr virus infection’ ‘Drug metabolism - cytochrome P450’, ‘Fc epsilon RI signaling pathway’, ‘Carbohydrate digestion and absorption’, ‘Chloroalkane and chloroalkene degradation’, ‘Naphthalene degradation’, ‘Degradation of aromatic compounds’, and ‘Chemokine signaling pathway’ (Fig. 3A). The DEPs in B3 vs. MTC were primarily enriched in ‘Complement and coagulation cascades’, ‘Ascorbate and aldarate metabolism’, and ‘Lysine degradation’ (Fig. 3B). The DEPs in B3 vs B1 were mainly enriched in ‘Cholesterol metabolism’ and ‘Steroid biosynthesis’ (Fig. 3C).

### The PPI network analysis

**Fig. 4:**
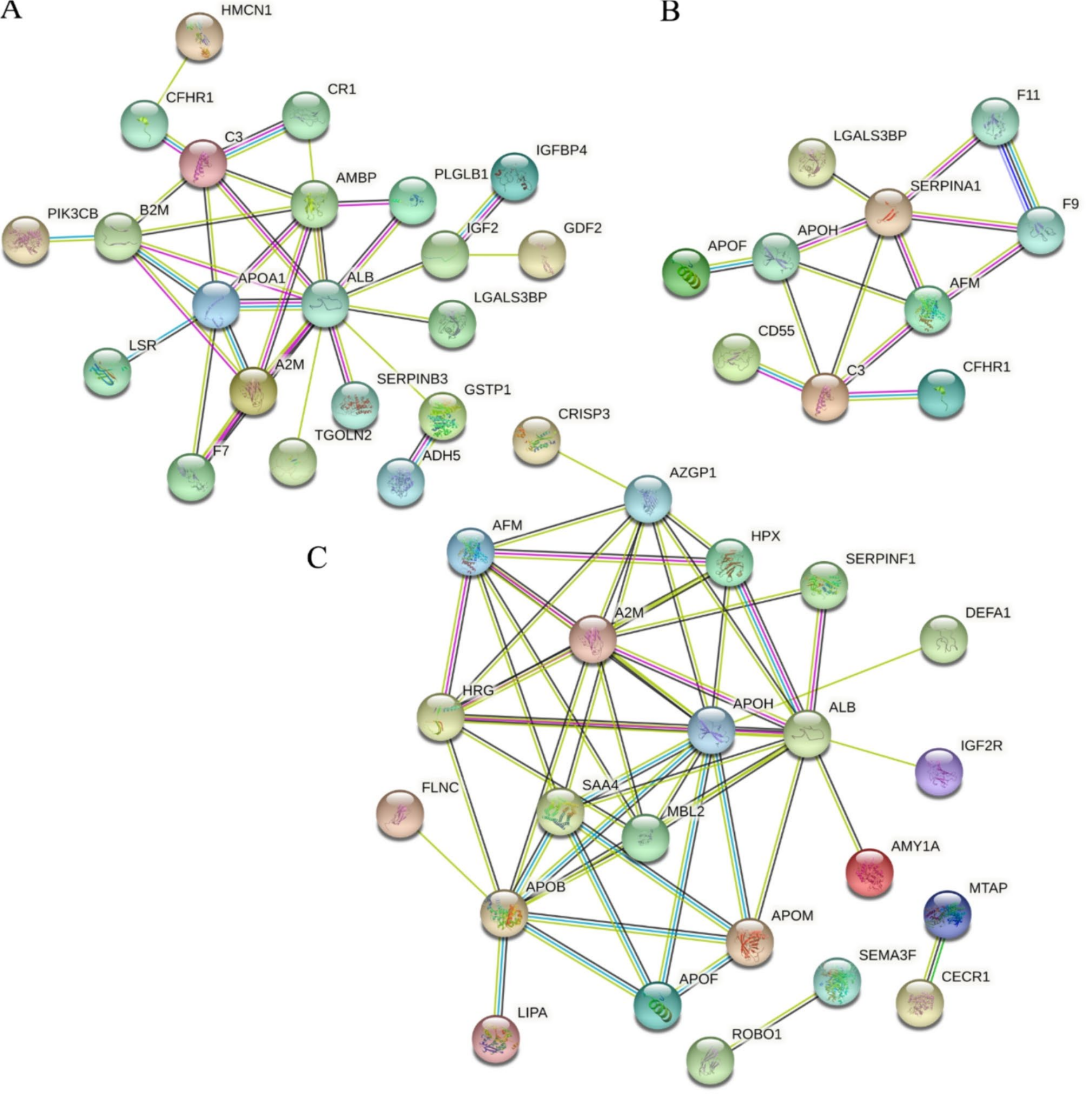
The PPI analysis of DEPs in B1 vs. MTC (**A**), B3 vs. MTC (**B**), and B3 vs. B1 (**C**) comparisons

As per the prediction data from GO and KEGG analyses, the PPI network of DEPs of B1 vs. MTC, B3 vs. MTC, and B3 vs. B1 was constructed. It is shown in Fig. 4A that C3, CR1, AMBP, ALB, APOA1, A2M, PLGLB1, and B2M interacted with each other in the B1 vs. MTC group. F11, F9, SERPINA1, LGALS3BP, APOH, C3, CD55, APOF, and CFHR1 were connected (Fig. 4B). As for B3 vs. B1, AFM, AZGP1, A2M, HPX, SERPINF1, ALB, APOH, SAA4, MBL2, APOB, and HRG closely interacted with each other (Fig. 4C).

### Key DEGs expression validation and UALCAN pan-cancer analysis

**Fig. 5:**
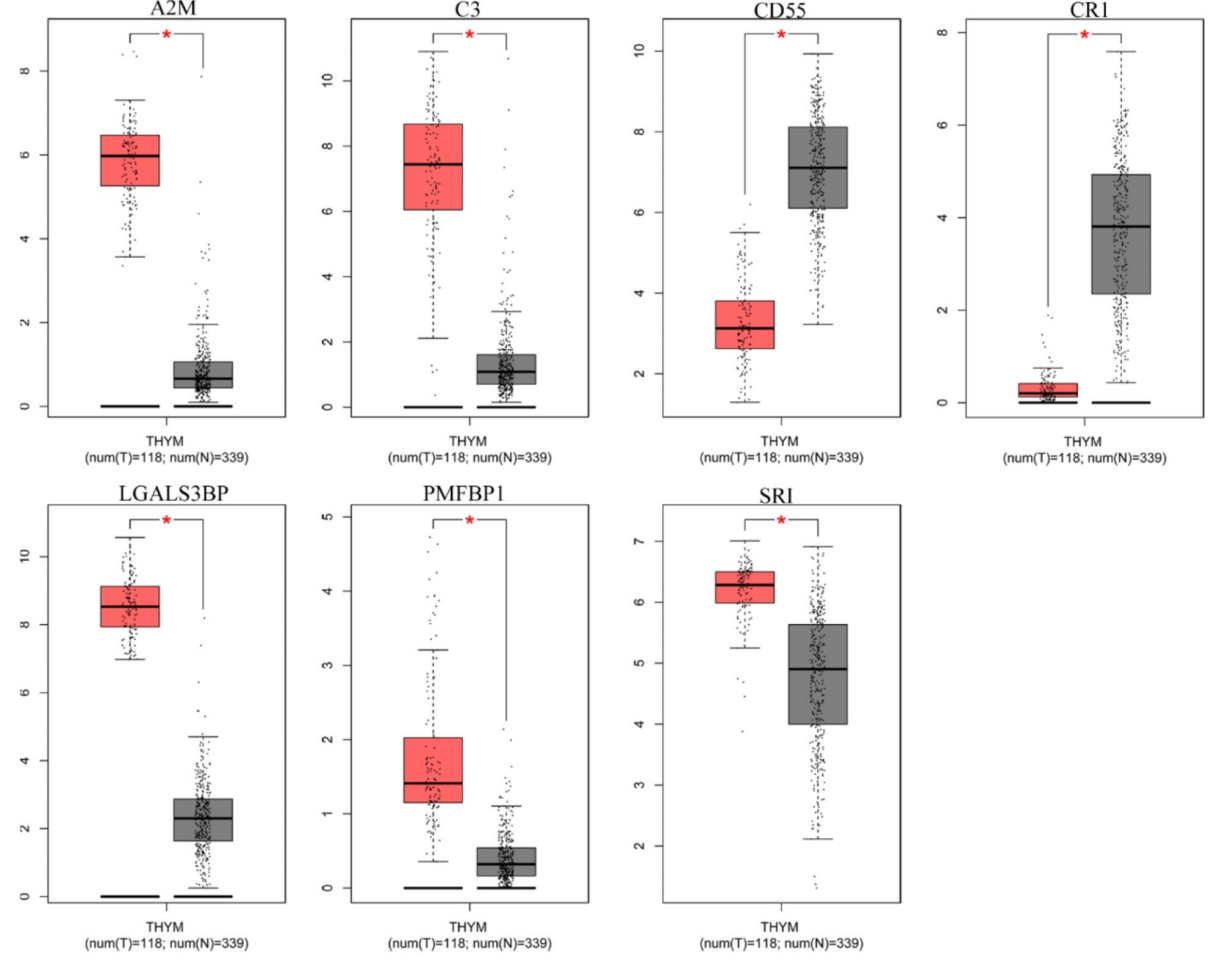
The expression level of DEGs in TCGA databases

**Fig. 6:**
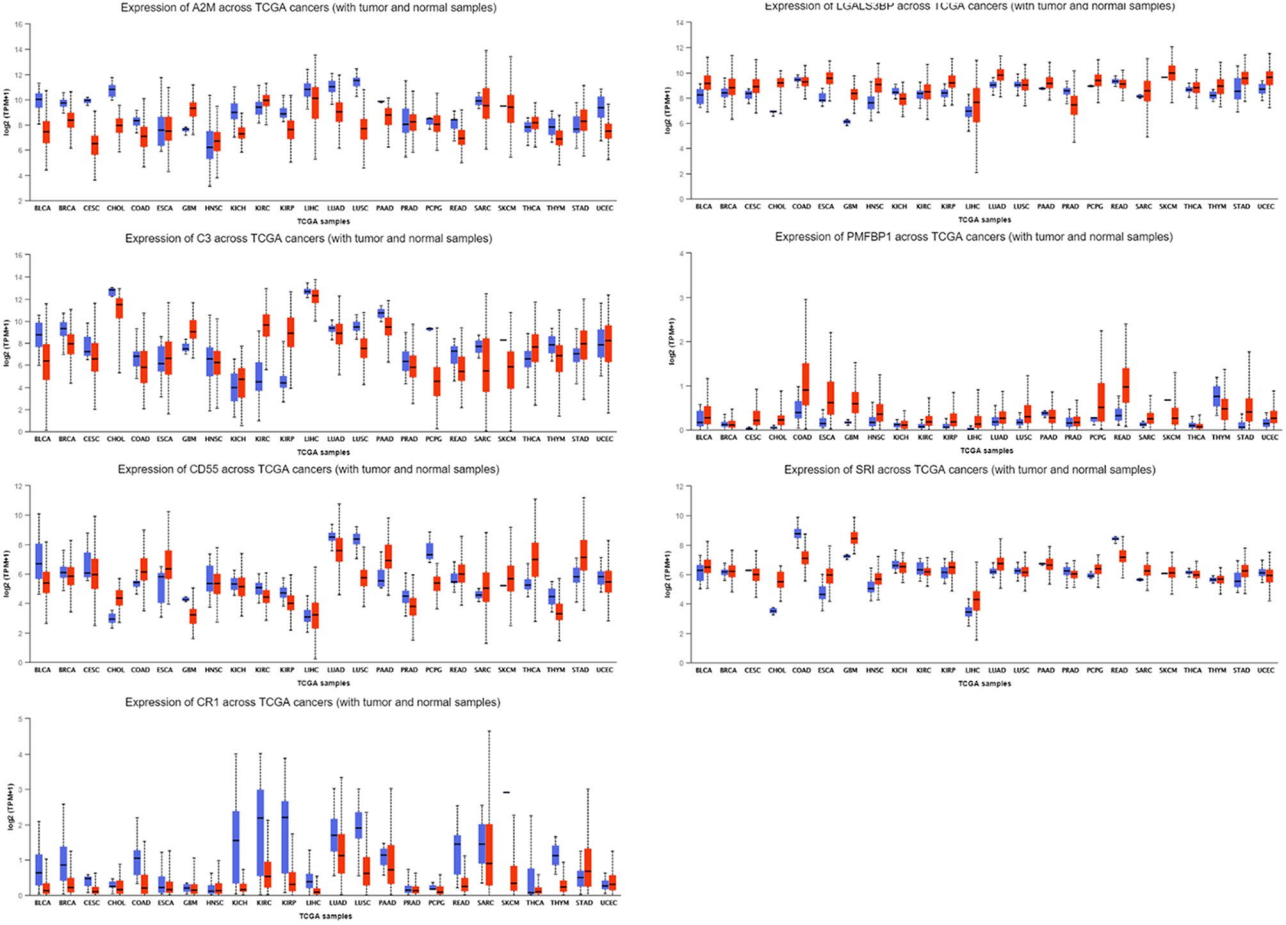
The expression of A2M, C3, LGALS3BP, PMFBP1, SRI, CD55 and CR1 in pan-cancers

GEPIA was employed to validate some of the critical DEGs (Fig. 5). It was discovered that A2M, C3, LGALS3BP, PMFBP1, and SRI were remarkably upregulated in individuals with thymoma (*P* < 0.05). CD55 and CR1 were considerably downregulated in thymoma patients. Surprisingly, the dysregulation trend of LGALS3BP is consistent with our study. Afterward, their expression was assessed in other cancers via UALCAN (Fig.  6). Generally, all these genes were significantly dysregulated in thymoma. Moreover, it was found that A2M has a moderate upregulation in almost all cancers except glioblastoma multiforme (GBM), head and neck squamous cell carcinoma (HNSC), and stomach adenocarcinoma (STAD). Despite dysregulation in certain cancers, the expression levels of CR1 and PMFBP1 remained at a superficial level. LGALS3BP exhibited relatively moderate expression in tumors and normal tissues.

## Discussion

The diagnosis of thymoma from a thymomaic cyst primarily relies on CT or surgery. Approximately half of all unnecessary thymectomy procedures were conducted due to concerns related to thymoma. Hence, the significance of valuable, non-invasive biomarkers is progressively on the rise. This research has identified B1 and B3 thymoma indicators that are distinct from thymic cysts. Through this investigation, this research aims to identify relevant biomarkers for clinical diagnostic purposes.

IL13RA1, LGALS3BP, PRCSH, C3, MXRA5, TNN, CFHR1, and SUN3 were common DEPs in both B1 vs. MTC and B3 vs. MTC comparisons. The GEPIA database verified that LGALS3BP is highly expressed in thymoma. LGALS3BP is a secreted multifunctional glycoprotein present in human body fluids. Glycoproteins are a class of proteins with glycans linked to amino acid side chains. These proteins play a significant role in cancer, as cell transformation often correlates with alterations in protein glycosylation [13]. The primary forms of protein glycosylation changes linked to tumor transformation involve modifications in O-glycans (GalNAc-Ser/Thr) and N-glycans [14]. Changes in protein glycosylation can lead to alterations in glycoprotein conformation, oligomerization, and turnover rates, and may also be associated with modifications in cellular signaling pathways, such as proliferation signals, resistance to cell death, evasion of growth suppression, genomic instability and mutations, angiogenesis, invasion and metastasis, pro-tumor inflammation, and immune evasion. Overexpression of LGALS3BP has been reported in various other carcinoma types. For instance, LGALS3BP is associated with malignant progression and a poor prognosis for CRC [15]. LGALS3BP increases oral squamous cell carcinoma cell proliferation and migration primarily via the PI3K/AKT pathway Similar conclusions were declared by Song et al. [16] in their in vitro and in vivo studies of endometrial cancer. It was demonstrated that upon LGALS3BP overexpression, the PI3K/AKT/VEGFA signaling pathway was activated, significantly promoting the proliferation and migration of endometrial cancer cells [17]. However, it has been suggested that LGALS3BP is associated with favorable clinical outcomes in colorectal cancer [18], pleural mesothelioma [19], and Ewing sarcoma [20]. The diverse pro-tumor mechanisms in which LGALS3BP plays a role are associated with its multi-domain structure and its interactions with various ligands, encompassing adhesion, migration, angiogenesis, motility, and the immune response. Recently, it has become clear that LGALS3BP associated with extracellular vesicles is a key regulator of intercellular and extracellular matrix crosstalk in the context of cancer [21]. In this present study, LGALS3BP has been found to be highly expressed in thymoma. Nonetheless, the intricate mechanisms of LGALS3BP involved in thymoma development and progression require further experiments.

Compared to B1 thymoma, the thymic cyst DEPs were enriched in ‘Drug metabolism -cytochrome P450’. A recent study found that genes implicated in a competing endogenous RNA network were dramatically upregulated during xenobiotic metabolism by cytochrome P450 in papillary thyroid cancer [21]. Zhong et al. [22] and Tang et al. [23] also demonstrated that the mRNAs of papillary thyroid carcinoma were enriched in ‘Drug metabolism - cytochrome P450,’ which was consistent with the findings of the current study. The ability of the cytochrome P450 enzyme to catalyze the metabolism of various endogenous and exogenous compounds is closely correlated to the development of tumors. Consequently, the development of drugs that target the cytochrome P450 enzyme has become one of the novel research directions in tumor therapy. In thymoma, treatments targeting cytochrome P450 require further investigation.

Regarding the identification of biomarkers between B1 and B3 thymoma, DEPs were primarily enriched for ‘cholesterol metabolism’ and ‘steroid biosynthesis.’ Evidence from the TCGA database also indicates that the cholesterol homeostasis pathway is implicated in the development of cancer. Alterations in gene expression levels and mutations associated with cholesterol homeostasis pathways have been identified in cancer cells [24]. These include increased gene copy numbers, upregulation of cholesterol synthesis gene expression, enhanced LDL receptor cholesterol import, and reduced cholesterol transport, which contribute to elevated cellular cholesterol levels to aid cancer cell proliferation [25]. Internal and external factors play a crucial role in regulating cholesterol metabolism and driving changes in the cholesterol pathway within cancer cells. Internal factors, including certain signaling pathway molecules and cholesterol itself, influence cholesterol metabolism by modulating the activities of SREBP and LXR. External factors, such as acidification and inflammation within the tumor microenvironment (TME), may also impact cholesterol metabolism [26]. In the tumor microenvironment, cell-intrinsic and cell-extrinsic cues reprogram cholesterol metabolism and enhance tumorigenesis.In addition, numerous studies indicate that ncRNAs influence cholesterol homeostasis by regulating cholesterol transport, uptake, and efflux, with these ncRNAs playing a pivotal role in cancer progression [27]. The reprogramming of lipid metabolism is a hallmark of cancer. Cholesterol, a vital component of lipids, is considered indispensable for the proliferation and survival of cancer cells. Mechanistically, cholesterol influences tumor cells through the regulation of immune response, iron condensation and autophagy, tumor cell stemness, and DNA damage response. Meanwhile, complex roles are played by cholesterol-derived metabolites in suppressing immune responses and promoting cancer progression. Based on both preclinical and clinical investigations, manipulating cholesterol metabolism has been shown to suppress tumor growth, alter the immunological environment, and enhance anti-tumor immune responses [28, 29]. However, numerous questions remain unanswered. For instance, which event occurs first: the disruption of cholesterol metabolism or the development of tumors? Conclusive evidence linking cholesterol and tumorigenesis is lacking. Cholesterol homeostasis is governed by intricate feedback loops, and these mechanisms within tumors remain unexplored. Given the complexity of the cholesterol metabolism network, inhibiting a single pathway in cholesterol metabolism may yield minimal therapeutic benefit against cancer. An increasing body of research indicates that the crosstalk between cholesterol metabolism-related factors and TME molecules is pivotal in tumor growth. Moreover, developing antitumor inhibitors that modulate TME and cholesterol levels is an attractive research strategy. Overall, cholesterol metabolism and cancer represent significant areas of focus in future anticancer research. As reported, the overall survival time of B1 was typically longer relative to B3 [30]. Given the insulin-dependent nature of thymoma [31], it is resonable to believe that targeting cholesterol metabolism might provide a potential benifits to patients [32]. However, the association between cholesterol metabolism and, steroid biosynthesis and overall survival time has yet to be investigated.

Furthermore, this study conducted a pan-cancer analysis on certain DEGs. These DEGs exhibited significant dysregulation across almost all cancer types, indicating their potential utility as biomarkers for malignancies. Nonetheless, it is noteworthy that none of these DEGs investigated emerged as a promising biomarker for thymoma.

## Conclusions

In this study, the biomarkers in B1, B3 thymoma, and thymic cysts were identified in the tissues of patients. It was determined that LGALS3BP might be an essential biomarker to identify thymoma from the thymic cyst. Targeting drug metabolism-cytochrome P450 is one potential therapy for B1 thymoma. Cholesterol metabolism might be necessary for classifying B1 and B3 thymoma.

## Data Availability

No datasets were generated or analysed during the current study.
